# Local adaptation mosaic to leaf herbivores in the annual herb *Datura stramonium*

**DOI:** 10.1007/s10265-025-01664-2

**Published:** 2025-08-29

**Authors:** Guillermo Castillo, Adán Miranda-Pérez, Ken Oyama, Juan Núñez-Farfán

**Affiliations:** 1 Facultad de Enología y Gastronomía, Universidad Autónoma de Baja California, Carretera Transpeninsular Ensenada-Tijuana 3917, Colonia Playitas, Ensenada, Baja California C.P. 22860 México; 2 Laboratorio de Genética Ecológica y Evolución, Departamento de Ecología Evolutiva, Instituto de Ecología, Universidad Nacional Autónoma de México, Circuito Exterior, Ciudad Universitaria, Distrito Federal, 14510 México; 3 Escuela Nacional de Estudios Superiores, Universidad Nacional Autónoma de México, Campus Morelia, Michoacán, México

**Keywords:** Coevolution, Generalist herbivores, Leaf trichome density, Natural selection, Plant resistance, Specialist herbivores

## Abstract

Local adaptation is a central evolutionary process for creating/maintaining variation of traits mediating antagonistic interactions. However, few studies have evaluated the local adaptation of plants to their biological counterparts such as herbivores across the plants’ distribution. Most studies evaluating local adaptation to herbivores have focused on specialist systems, where local adaptation is likely to occur. However, there is less evidence regarding the existence of local adaptation on generalist systems, where local adaptation is not theoretically expected. We conducted a reciprocal transplant experiment involving four local populations aimed to detect whether local adaptation in the annual herb *Datura stramonium* to its specialist herbivore *Lema daturaphila* and the generalist herbivore *Sphenarium purpurascens* occur. We also explored whether leaf trichome density, a putative defensive trait of *D. stramonium*, is mediating local adaptation to herbivores through its association with plant fitness. We found that certain *D. stramonium* populations were locally adapted to both herbivores but others were not, regardless of whether these are preyed upon by generalist or specialist herbivores. Leaf trichome density had a significant effect on individual fruit production, although this effect was variable across locations (origin × site interaction) and unrelated to the observed local adaptation pattern. The results support a view of a local adaptation mosaic of *D. stramonium* to generalist and specialist herbivores in central Mexico.

**Supplementary Information:**

The online version contains supplementary material available at 10.1007/s10265-025-01664-2.

## Introduction

Species are usually considered groups of populations genetically and phenotypically differentiated (Rice and Jain 1985; Thompson 2005). Such differentiation may arise through neutral processes such as interrupted gene flow, genetic drift or founder effects (Gomulkiewicz et al. 2007), or it may result from spatial variation in selection pressures exerted by biotic and abiotic factors (Holsinger and Weir 2009; Lande and Arnold 1983; Nuismer and Gandon 2008). Uncovering the relative role of these forces in generating and maintaining phenotypic variation constitutes a main goal of evolutionary ecology (Leinonen et al. 2006, 2013; Mazer and Damuth 2001).

Local adaptation studies reveal the role of selection in generating divergence in traits to mediate adaptive fit to local conditions (Vesakoski and Jormalainen 2013; Wadgymar et al. 2022). According to Kawecki and Ebert (2004), local adaptation is detected when in a given habitat, individuals from the local population show higher fitness compared to individuals originating from other habitats (local vs. foreign criterion). Local adaptation can also be detected when individuals of a population show higher fitness in their own habitat than in other habitats (home vs. away criterion). However, it has been suggested that the “home vs. away” criterion alone does not provide enough evidence of local adaptation, because it may reflect differences in habitat quality rather than the effects of divergent selection (DeFaveri and Merilä 2014; Kawecki and Ebert 2004). Blanquart et al. (2013) proposed a third criterion to detect local adaptation, namely the sympatric vs. allopatric contrast. Under this approach local adaptation should be considered a property of a metapopulation (rather than a property of a single population) that can be measured as the fitness differences between populations in their home sites (sympatry) compared with foreign sites (allopatry).

Several studies in plants have explored local adaptation to abiotic conditions (see Leimu and Fischer 2008). However, fewer studies have evaluated plants’ local adaptation to their biological enemies (Garrido et al. 2012; Laukkanen et al. 2012; Ortegón-Campos et al. 2009). In the case of plant-herbivore interactions, local adaptation is detected when plants originated from a population exhibit higher fitness in relation to their sympatric herbivores than when exposed to allopatric herbivores (Kawecki and Ebert 2004; Laukkanen et al. 2012). Most evidence supporting this hypothesis arises from specialist interaction systems, where interacting species are engaged in a coevolutionary arm-races, and local adaptation is likely to occur (Gandon and Michalakis 2002). In contrast, less evidence is available for generalist systems, where multi-species selection, if it occurs, is the norm and where local adaptation is not predicted (Gómez et al. 2009; Ortegón‐Campos et al. 2009). Furthermore, since leaf damage by herbivores generally has a negative impact on plant fitness (Crawley 1989), it is expected that local adaptation to herbivores would be mediated by herbivores´ feeding preferences and by the defensive traits exhibited by plants (Laukkanen et al. 2012).

The annual herb *Datura stramonium* L. is an ideal system to test local adaptation to dietary generalist and specialist herbivores. Mexican populations of *D. stramonium* are attacked mainly by the specialist folivore *Lema daturaphila* (Chrysomelidae) (Nuñez-Farfan and Dirzo 1994; Valverde et al. 2001). In contrast, *L. daturaphila* is absent in some populations, making the oligophagous *Epitrix parvula* (Chrysomelidae) and the generalist *Sphenarium purpurascens* (Orthoptera) the main plant consumers (Castillo et al. 2014). Leaf trichome density in *D. stramonium* functions as a defensive trait against herbivory, and it may evolve by selection exerted by herbivores (Kariñho-Betancourt and Núñez-Farfán 2015; Valverde et al. 2001). Likewise, populations of *D. stramonium* show geographic variation in the abundance of tropane alkaloids, leaf trichome density, herbivory, and plant fitness (Castillo et al. 2013; De-la-Cruz et al. 2020a).

Previous studies have detected a natural selection mosaic acting on resistance traits across populations of *D. stramonium* in central Mexico (Castillo et al. 2014). Thus, the adaptive value of a defensive trait such as leaf trichome density may vary from negative (phagostimulant to herbivores) to positive (herbivore deterrent) across populations (Kariñho-Betancourt and Núñez-Farfán 2015; Valverde et al. 2001). In a greenhouse experiment, Garrido et al. (2012) detected local adaptation of *D. stramonium* to its specialist herbivore *L. daturaphila*. De-la-Cruz et al. (2020b) assessed how spatially variable selection operates on the genetic basis of plant defenses to herbivores in *D. stramonium*, through genomic and quantitative genetic approaches. Their results showed that specific alkaloids and their associated loci/alleles were favored by selection imposed by different herbivores, leading to population differentiation in plant defenses and to local adaptation driven by plant-herbivore interactions. Recently, (De-la-Cruz et al. 2024) studied the genetic architecture of plant resistance to insect herbivores of *D. stramonium* through quantitative trait loci mapping. Results indicated that a single quantitative trait loci region on chromosome 3 of *D. stramonium* is associated with plant resistance. This QTL explained 8.44% of the phenotypic variance in plant resistance. Receptor-like protein kinases were found to play a pivotal role in controlling signaling transduction pathways associated with the biosynthesis of secondary compounds (phenylalanine, terpenoids, carotenoids, and tropane alkaloids) within the QTL region. Furthermore, a protein from the TRICHOME BIREFRINGENCE-LIKE, responsible for trichome productions in plants was also identified within the QTL region. Yet, it remains unknown whether *D. stramonium* populations can also become adapted to generalist herbivores, and whether a physical resistance trait, such as leaf trichome density, can mediate local adaptation to herbivores. Here, we conducted a reciprocal transplant experiment between four populations of *D. stramonium* aimed to (*i*) detect local adaptation to specialist and generalist herbivores, and (*ii*) to assess whether local adaptation to herbivores is mediated by leaf trichome density.

We expect that *D. stramonium* shows local adaptation in populations preyed upon by the specialist herbivore *L. daturaphila* and that this adaptation is absent in populations where the generalist herbivore *S. purpurascens* is predominant. Nevertheless, we expect that leaf trichome density, because of its correlation with plant fitness, confers some degree of resistance against both specialist and generalist herbivores (cf. Castillo et al. 2013; Kariñho-Betancourt and Núñez-Farfán 2015; Valverde et al. 2001).

## Materials and methods

### Study system

*D. stramonium* L. (Solanaceae) is an annual subcosmopolitan herb commonly found nearby cultivated areas and disturbed environments in Mexico, the United States, Canada, and Europe (Shonle and Bergelson 2000; Valverde et al. 2001; Weaver and Warwick 1984). The species reproduces mainly by self-fertilization, and has limited pollen and seed dispersal (Motten and Antonovics 1992). Previous studies have shown that *D. stramonium* has a moderate genetic structure in Central Mexico, *R*_ST_ = 0.228 (Castillo et al. 2015). Most populations of *D. stramonium* in Mexico are consumed by the specialist folivorous beetle *L. daturaphila* (Nuñez-Farfan and Dirzo 1994). However, there are populations where *L. daturaphila* is absent, and where the oligophagous flea beetle *E. parvula* (which consumes other members of the Solanaceae family), and/or the generalist grasshopper *S. purpurascens*, are the main consumers of the plants (Castillo et al. 2014). Extensive surveys of populations of *D. stramonium* in Mexico, analyzing the levels of parasitoidism of *L. daturaphila*´s eggs by the micro wasp *Emersonella lemae* (Villanueva-Hernández and Núñez-Farfán 2025), point out that *L. daturaphila* co-occurs with *D. stramonium* mostly in temperate places, most above 2000 m.a.s.l. The Trans Mexican volcanic belt, that runs from Nayarit in the Pacific Coast up to the coasts of the Gulf of Mexico in Veracruz, marks the southern distribution limit of *L. daturaphila* whose distribution extends up to the Northeast of the USA (J. Núñez-Farfán, Personal Observations).

Leaf damage exerted by each of these herbivores is characteristic and recognizable (a more detailed description of leaf damage type by each herbivore can be found elsewhere (Carmona and Fornoni 2013; Castillo et al. 2014; Nuñez-Farfan and Dirzo 1994). Previous studies on *D. stramonium* have shown that leaf trichome density constitutes a resistance trait against herbivory that possess genetic variance (Kariñho-Betancourt and Núñez-Farfán 2015; Valverde et al. 2001).

### Reciprocal transplants experiment

**Fig. 1:**
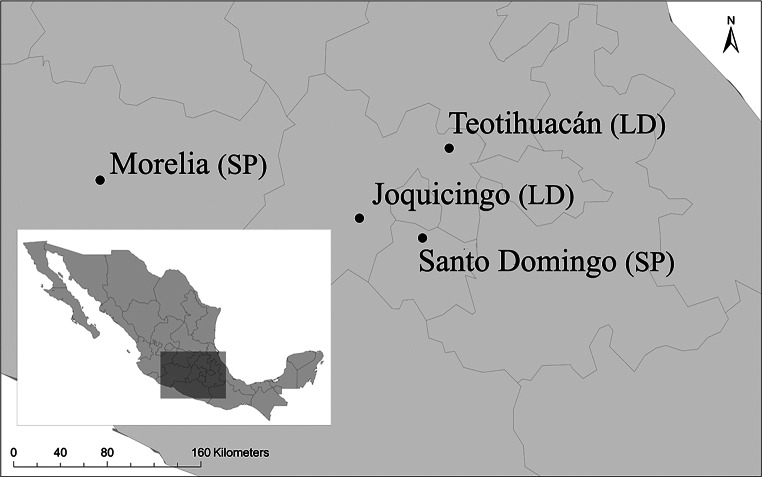
Location of four populations of *Datura stramonium* selected to perform a reciprocal transplant experiment to detect local adaptation. The Teotihuacán and Joquicingo populations are primarily consumed by the specialist herbivore *Lema daturaphila* (LD), although they are also fed upon by the generalist *Sphenarium purpurascens* (SP). In Morelia and Santo Domingo populations the plants are mainly consumed by the generalist *S. purpurascens*; here the specialist *L. daturaphila* was present at low abundance

During September-November 2013 we conducted reciprocal transplant experiments between plants originated from four different natural populations of *D. stramonium* (Fig. 1). Populations were selected considering the presence of the main herbivore at each locality. Teotihuacán and Joquicingo populations are primarily consumed by the specialist herbivore *L. daturaphila*, although they are also fed upon by the generalist *S. purpurascens*. Conversely, Morelia and Santo Domingo populations are mainly consumed by the generalist *S. purpurascens*, with the specialist *L. daturaphila* also present but at a low frequency during the experiment. Although the abundance of herbivores in *D. stramonium* populations can vary interannually, likely reflecting climatic variation, their presence remains predictable across years, and the predominant herbivore species persist at each population (Castillo et al. 2014; De-la-Cruz and Núñez-Farfán 2023). Distances between populations ranged from 70 to 290 km. Geographic coordinates, climatic conditions and habitat characteristics of each population are shown in Table S1.

Experimental plants were obtained by germinating seeds collected from 30 mother plants from each population of origin. Because a single fruit can produce more than 200 seeds, we germinated seeds from a single fruit from each mother plant (i.e., full-sibs). Seeds were individually germinated in plastic pots containing a commercial soil mix in a glasshouse. This process allowed us to generate four groups of 30 plants, each corresponding to one of the four sites of origin. Once the plants produced their first true leaves, they were transplanted into four experimental plots at each site of origin in a fully randomized block design. In order to separate biotic and abiotic effects on plant fitness at each experimental site, we included an herbivore exclusion treatment. For this, we applied (1 g/50 ml) the commercial systemic insecticide Furadan (FMC Corporation, Philadelphia, PA, USA).

### Leaf damage

The proportion of leaf damage removed by herbivores per plant was calculated as the ratio of leaf area removed by herbivores in a sample of 20 leaves divided by the total area of those same 20 leaves. When a plant had less than 20 leaves, we collected all of them. We predicted total area of a given sampled leaf, by using a regression of leaf area as a function of leaf length from a sample of undamaged leaves (Valverde et al. 2001). We used a separate regression model for each population to account for differences in leaf shape among populations of different origin (*R²* ranged from 0.934 to 0.958, *p* < 0.001, *n* = 30). The remaining leaf area was measured using the software Image-Pro Plus [Version 7.0 for Windows; Media Cybernetics Inc., Bethesda, MD, USA].

### Trichome density and plant fitness

We estimated leaf trichome density as the total number of trichomes in an observation field of 2.5 mm^2^ located in the central basal region of the adaxial side of the leaf, following Valverde et al. (2001). We obtained the trichome density per plant, averaging trichome number of a random sample of 20 fully expanded leaves. We used the number of fruits per plant as a proxy for individual maternal fitness. Since *D. stramonium* is an annual selfing plant, fruit volume is highly correlated with the total number of seeds contained in a fruit (Fornoni et al. 2003). Therefore, the number of fruits is a reliable proxy for lifetime fitness (Mauricio and Rausher 1997).

### Statistical analyses

#### Herbivore exclusion

To evaluate whether herbivore exclusion treatment was efficient in preventing/reducing leaf damage, we used a multifactorial ANOVA with the terms insecticide, experimental site as well as the insecticide × experimental site interaction as predictor variables, and the proportion of leaf damage as response variable. Leaf damage proportion was log-transformed prior to analyses. Similarly, we evaluated the effect of the insecticide treatment on fruit production using a quasi-poisson GLM with a log link function. Predictor variables used in this model were the same as described above but using fruit production as response variable.

#### Local adaptation

We tested for local adaptation of *D. stramonium* to generalist and specialist herbivores by fitting a negative binomial generalized linear model (GLM) with a log link function. This analysis was conducted on a subset of the data that excluded plants from the herbivore-exclusion treatment. The model included the terms population origin (local vs. foreign), experimental site, and trichome density, plus all two- and three-way interactions. The interaction Site × trichome density tests whether the defensive value of trichomes depends on the local herbivore community, whereas Origin × trichome density and Origin × Site × trichome density assess whether any detected local adaptation is mediated by variation in this trait. Whenever a significant interaction origin × experimental site was detected (first requisite for detecting local adaptation) we carried out directed linear contrasts for testing the (i) “local vs. foreign”, (ii) “home vs. away” and (iii) “sympatric vs. allopatric” criterions of local adaptation, as described in Blanquart et al. (2013).

#### Leaf trichome density contribution to local adaptation

To evaluate if trichome density accounts for the local adaptation of *D. stramonium* to generalist and specialist herbivores, we tested the correlation between leaf trichome density and fruit number using simple Spearman’s rank correlations (*ρ*) for each population at each experimental site, using a subset of the data that excluded the herbivore exclusion treatment. All analyses were performed in R statistical software version 3.0.2 (R Development Core Team 2011).

## Results

### Herbivore exclusion

We found a significant effect of the insecticide treatment on leaf damage. Overall efficiency of the insecticide treatment was 58.82% (in terms of leaf damage reduction). We also found a significant effect of the term experimental site (Table 1a). Mean leaf damage reduction at each experimental site was: Joquicingo 61.11%, Teotihuacán 55.11%, Santo Domingo 48.81% and Morelia 46.81%. A significant effect of the experimental site × insecticide interaction was also detected (Table 1a) (Fig. S1). On the other hand, herbivore removal treatment increased on average 25.4% of fruit production (Table 1b). Significant differences in fruit number among experimental sites were also detected (Table 1b). Finally, a significant experimental site × insecticide interaction was detected (Table 1b) (Fig. S2).

**Table 1:** Results of (**a**) an ANOVA model analyzing the effects of insecticide, site, and their interaction (Insecticide × Site) on leaf damage, and (**b**) a GLM model assessing the effects of insecticide, site, and their interaction on fruit production, in a reciprocal transplant experiment of *Datura stramonium*

Response variable	Term	d. f.	F	*p*	*R* ^2^
a) Leaf damage	Insecticide	1	65.53	**< 0.0001**	0.23
	Site	3	48.02	**< 0.0001**	
	Insecticide × Site	3	2.93	**0.0046**	
	Error	841	188.47		
	Total	848	321.52		
	**Term**	***d. f.***	***χ*** ^***2***^	***p***	
b) Fruit number	Insecticide	1	5.69	**0.017**	
	Site	3	370.02	**< 0.0001**	
	Insecticide × Site	3	33.64	**< 0.0001**	

Significant values are highlighted in bold

### Local adaptation

We found a significant effect of the term experimental site on fruit production (Table 2). Mean fruit production was 3.78 in Joquicingo, 7.8 in Teotihuacán, 2.1 in Santo Domingo and 9.38 in Morelia. A significant effect of trichome density was also detected (Table 2). Moreover, the experimental site × trichome density and origin × trichome density interactions were significant (Table 2). Likewise, we detected a significant effect of the interaction experimental site × origin (Table 2).

**Table 2:** GLM model of the effects of population of origin, experimental site (i.e., “Site”) and leaf trichome density on fruit production of *Datura stramonium*

Variable	d. f.	F	*p*
Site (S)	3	62.0781	**< 0.001**
Origin (O)	3	1.4951	0.2
Trichome Density (TD)	1	14.892	**< 0.001**
S × O	9	7.3266	**< 0.001**
S × TD	3	3.8664	**0.009**
O × TD	3	3.0473	**0.03**
S × O × TD	9	1.3427	0.2

Significant values appear in bold-type font

**Fig. 2:**
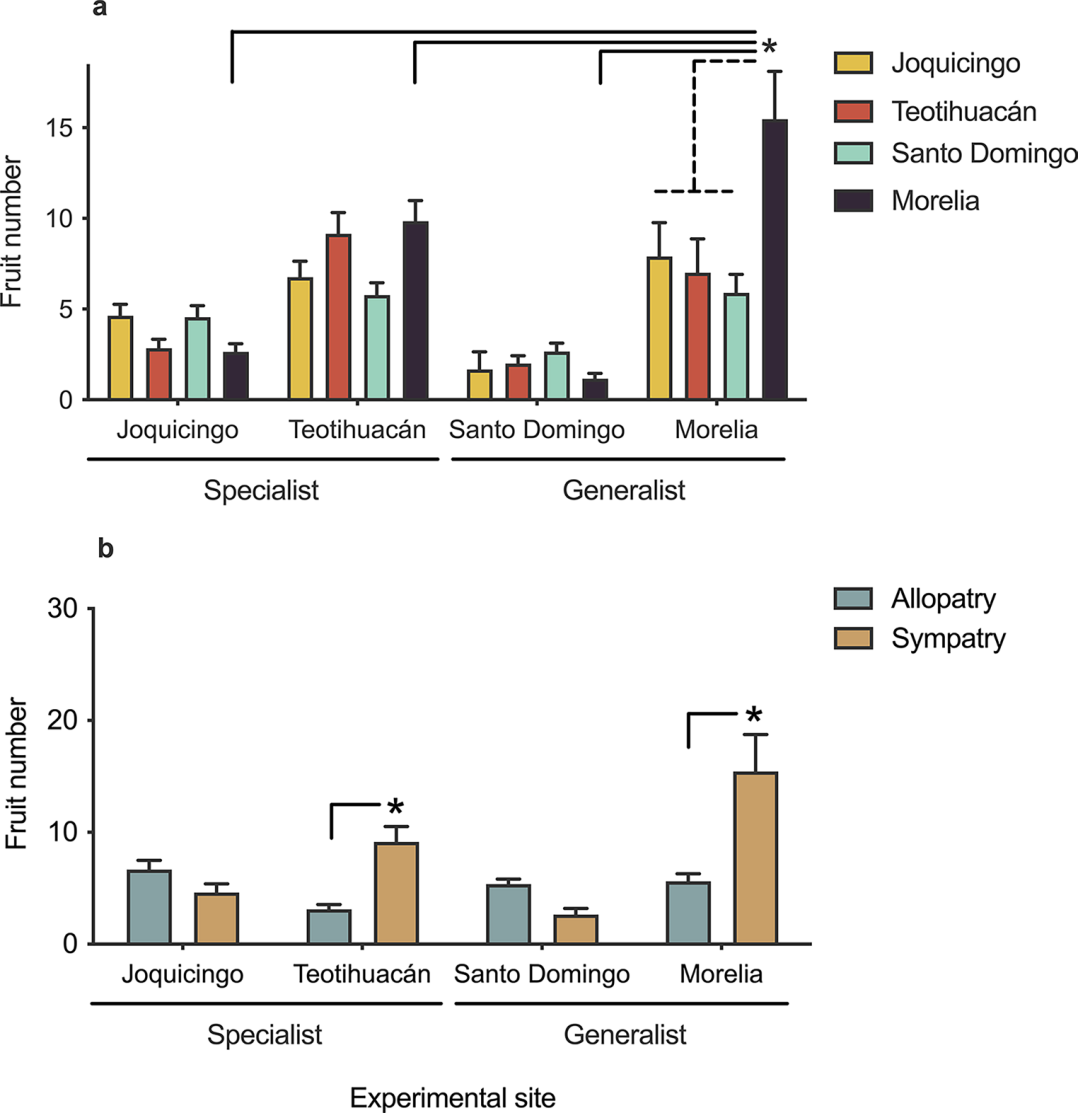
Analyses of *Datura stramonium* plants exposed to herbivores show evidence of local adaptation for the Morelia population to the generalist herbivore *Sphenarium purpurascens*. **a**) Mean fruit production of four populations of *D. stramonium* in a reciprocal transplant experiment. The asterisk indicates significant differences (*p* < 0.05) of linear contrasts comparing fruit production according to the “local vs. foreign” (dashed line) and “home vs. away” (continuing lines) criteria for testing local adaptation. **b**) Mean fruit production in allopatric and sympatric combinations of experimental sites and populations of origin. The asterisk indicates significant differences (*p* < 0.05) of linear contrasts comparing fruit production according the “sympatric vs. allopatric” criterion for testing local adaptation. Error bars represent ± 1 SE

Direct contrasts aimed to detect local adaptation according the local vs. foreign criterion (i.e., higher mean fitness of a focal population at home, in comparison to the average mean fitness of all the other populations when transplanted into the focal population) indicated the existence of local adaptation for plants originated in Morelia population (Fig. 2a, dotted lines). Accordingly, the home vs. away criterion (higher mean fitness of the population at home compared to the average mean fitness of the same population when transplanted in all other habitats) also showed evidence for local adaptation for the Morelia population (Fig. 2a, solid line). Finally, the sympatric vs. allopatric criterion (higher mean fitness in sympatric combinations of populations and sites compared to average fitness in allopatric combinations) showed evidence of local adaptation in Teotihuacán and Morelia populations (solid lines Fig. 2b).

### Leaf trichome density contribution to local adaptation

Under the presence of herbivores, we found significant correlations between trichome density and fruit production in the Santo Domingo (*r* = -0.55, *p* = 0.0024) and Morelia (*r* = -0.6, *p* = 0.0002) populations in the Joquicingo experimental site. We also found significant correlations in the Santo Domingo population in the Santo Domingo experimental site (*r* = -0.41, *p* = 0.0336) and in the Santo Domingo population in the Teotihuacan experimental site (*r* = -0.52, *p* = 0.0002). Under herbivore-exclusion conditions, at the Joquicingo experimental site, plants originating from Santo Domingo showed a highly significant negative correlation (*r* = − 0.61, *p* = 0.0008), whereas plants originating from Teotihuacán exhibited a significant positive correlation (*r* = 0.49, *p* = 0.0082). At the Morelia experimental site, trichome density showed a negative correlation with fruit production in plants originating from Joquicingo (*r* = − 0.46, *p* = 0.0205), while plants originating from Santo Domingo displayed a significant positive correlation (*r* = 0.50, *p* = 0.0360).

## Discussion

Local adaptation is a fundamental process for creating and maintaining trait variation of antagonistic interactions (Laukkanen et al. 2012). However, few studies have evaluated local adaptation of plants to their biological counterparts (Leimu and Fischer 2008). Most of the empirical evidence of local adaptation to herbivores come from specialist systems, where local adaptation is likely to occur (Gómez et al. 2009; Ortegón-Campos et al. 2009). Yet, there is scarce evidence regarding the existence of local adaptation on generalist systems, were local adaptation is, in principle, not expected (Gómez et al. 2009). Here, we evaluated local adaptation of *D. stramonium* to its generalist and specialist herbivores. Our results indicate that *D. stramonium* can be locally adapted to both generalist and specialist herbivores but also indicate that it is not a generalized feature across populations. Since local adaptation to herbivores can depend on the feeding capacities of the herbivores, we also explored whether leaf trichome density (a defensive trait of *D. stramonium*) is mediating local adaptation to herbivores through correlations with fruit production. In this line, we found that trichome density indeed had a significant effect on individual fruit production, however we did not find a consistent pattern indicating that trichome density accounts for the observed local adaptation patterns. Our results suggest that this trait may even be costly.

To determine whether our estimates truly reflect local adaptation to herbivores, we incorporated an herbivore exclusion treatment in our reciprocal transplant experiment. Although we cannot rule out the influence of climatic factors, we expected higher fruit production in plants protected from herbivores compared to those fully exposed. Insecticide application significantly reduced herbivory rates, although it did not completely eliminate herbivory. Furthermore, fruit production was significantly higher under the low herbivore damage treatment than in plants fully exposed to herbivores, highlighting the negative impact of herbivory on fruit number production. However, the observed variation in the effectiveness of herbivore exclusion on fruit production across sites and populations suggests that local abiotic conditions may also play a role in shaping fruit production. Interestingly, the sign and strength of the correlation between trichome density and fruit production varied across experiments and herbivore-exclusion treatments, indicating that the role of trichome density results from the interplay among population origin, local herbivore pressure, and site-specific abiotic conditions.

Analysis of damage and performance of plants exposed to herbivores show evidence of local adaptation of *D. stramonium* for the Morelia population. In this site, the main leaf consumer was the generalist herbivore *S. purpurascens*. This population fulfills the three local adaptation criteria (see Fig. 2). Although local adaptation to generalist interactors is not generally expected in theory (Gómez et al. 2009), diverse studies of *D. stramonium* have detected selection by generalist herbivores on defensive traits (Castillo et al. 2014). On the other hand, we also found evidence of local adaptation in the Teotihuacán population to the specialist herbivore *L. daturaphila*, but only with the sympatric and allopatric contrast. In the local versus foreign contrast, the Teotihuacán population produced fewer fruits than the Morelia population during the experiment, although this difference was not statistically significant. This agrees with previous studies that observed mutual local adaptation of *D. stramonium* to *L. daturaphila* in a greenhouse setting (Garrido et al. 2012; Parachnowitsch and Lajeunesse 2012). However, our findings contrast with field experiments showing that the Teotihuacán population has lower resistance compared to the foreign Ticumán population and that selection exerted by *L. daturaphila* varies between years, with strong selection when *L. daturaphila* is more abundant (i.e., outbreaks; De-la-Cruz and Núñez-Farfán 2023). Furthermore, a recent study by Núñez-Farfán et al. (2024) found through a common garden experiment that native Teotihuacan plants had significantly more damage from *L. daturaphila* than to *D. stramonium* populations that have been introduced to regions outside their original native range, such as in Spain, indicating potential fitness differences. This may reflect, in turn, local adaptation of herbivores to their home host plant. Interestingly, the pattern of local adaptation to both generalist and specialist herbivores does not seem to be a generalized feature among *D. stramonium* populations. Instead, it resembles a geographic mosaic of local adaptations/maladaptations as predicted by the Geographic Mosaic Theory of Coevolution (Thompson 2001, 2005). Nevertheless, abiotic conditions, the availability of alternative host plants, or fluctuations in herbivore abundance may also affect the selective pressures shaping the resistance traits of *D. stramonium* (De-la-Cruz and Núñez-Farfán 2023).

We also explored whether trichome density accounts for local adaptation of *D. stramonium*. Here, we did not find a consistent pattern indicating that leaf trichome density is mediating local adaptation of *D. stramonium*. These results contrast with previous findings that detected strong selective pressures by herbivores acting on leaf trichome density (Kariñho-Betancourt and Núñez-Farfán 2015; Valverde et al. 2001). Castillo et al. (2015) found that trichome density is not adaptively differentiated in central Mexico, suggesting that most of the phenotypic variation of this trait could be related to other environmental factors, like mean precipitation and/or temperature (see Kariñho-Betancourt and Núñez-Farfán 2015). Furthermore, Castillo et al. (2014), detected correlational selection by specialist herbivores on trichome density and tropane alkaloids in *D. stramonium* populations in central Mexico, which suggests that these traits are part of a complex defensive syndrome (Agrawal and Fishbein 2006; Leimu and Koricheva 2006). Recent genetic studies found genomic regions in *D. stramonium* linked to resistance to herbivores that may be associated with the evolution of adaptive defense in this species (De-la-Cruz et al. 2024).

Unveiling the forces that maintain and generate phenotypic variation is a main goal for evolutionary biologists (Leinonen et al. 2006, 2013; Mazer and Damuth 2001). Here, we provide evidence suggesting that a plant species can locally adapt to both specialist and generalist herbivores. Our results also support the hypothesis that adaptive processes involved in plant-herbivore interactions are spatially structured. It is also worth mentioning that, in this study, we were not able to deal with other sources of variation such as, transgenerational induction and epigenetic inheritance of plant defensive traits. Several studies have found that herbivore and pathogen attack of plants can generate particular defense phenotypes across generations (Hannan Parker et al. 2022; Holeski et al. 2012; Kim and Felton 2013). Generating this kind of evidence was beyond the scope of this research, but including explicitly these factors in the context of the local adaptation could help us to explain rapid adaptation to herbivores on an ecological time scale.

## Supplementary Information

Below is the link to the electronic supplementary material.

Supplementary Material 1

## References

[CR1] Agrawal AA, Fishbein M (2006) Plant defense syndromes. Ecology 87:S132–S149. 10.1890/0012-9658(2006)87[132:PDS]2.0.CO;2 16922309 10.1890/0012-9658(2006)87[132:pds]2.0.co;2

[CR2] Blanquart F, Kaltz O, Nuismer SL, Gandon S (2013) A practical guide to measuring local adaptation. Ecol Lett 16:1195–1205. 10.1111/ele.12150 23848550 10.1111/ele.12150

[CR3] Carmona D, Fornoni J (2013) Herbivores can select for mixed defensive strategies in plants. New Phytol 197:576–585. 10.1111/nph.12023 23171270 10.1111/nph.12023

[CR4] Castillo G, Cruz LL, Hernández-Cumplido J, Oyama K, Flores-Ortiz CM, Fornoni J, Valverde PL, Núñez-Farfán J (2013) Geographic association and Temporal variation of defensive traits and leaf damage in *Datura stramonium*. Ecol Res 28:663–672. 10.1007/s11284-013-1059-4

[CR5] Castillo G, Cruz LL, Tapia-López R, Olmedo-Vicente E, Carmona D, Anaya-Lang AL, Fornoni J, Andraca-Gómez G, Valverde PL, Núñez-Farfán J (2014) Selection mosaic exerted by specialist and generalist herbivores on chemical and physical defense of *Datura stramonium*. PLoS ONE 9:e102478. 10.1371/journal.pone.0102478 25051169 10.1371/journal.pone.0102478 PMC4106780

[CR6] Castillo G, Valverde PL, Cruz LL, Hernández-Cumplido J, Andraca-Gomez G, Fornoni J, Sandoval-Castellanos E, Olmedo-Vicente E, Flores-Ortiz CM, Nunez-Farfán J (2015) Adaptive divergence in resistance to herbivores in *Datura stramonium*. PeerJ 3:e1411. 10.7717/peerj.1411 26644970 10.7717/peerj.1411 PMC4671194

[CR7] Crawley MJ (1989) Insect herbivores and plant population dynamics. Ann Rev Entomol 34:531–562

[CR10] De-la-Cruz IM, Núñez-Farfán J (2023) Inter-annual variation in the abundance of specialist herbivores determines plant resistance in *Datura stramonium*. Ecol Evol 13:e10794. 10.1002/ece3.10794 38077505 10.1002/ece3.10794 PMC10700045

[CR8] De-la-Cruz I, Cruz L, Martínez-García L, Valverde P, Flores-Ortiz C, Hernández-Portilla L, Núñez-Farfán J (2020a) Evolutionary response to herbivory: population differentiation in microsatellite loci, tropane alkaloids and leaf trichome density in *Datura stramonium*. Arthropod-plant Interact 14:21–30. 10.1007/s11829-019-09735-7

[CR9] De-la-Cruz IM, Merilä J, Valverde PL, Flores-Ortiz CM, Núñez-Farfán J (2020b) Genomic and chemical evidence for local adaptation in resistance to different herbivores in *Datura stramonium*. Evolution 74:2629–2643. 10.1111/evo.14097 32935854 10.1111/evo.14097

[CR11] De-la-Cruz IM, Oyama K, Núñez-Farfán J (2024) The chromosome-scale genome and the genetic resistance machinery against insect herbivores of the Mexican toloache, *Datura stramonium*. G3: Genes, Genomes, Genetics 14: jkad288. 10.1093/g3journal/jkad288 10.1093/g3journal/jkad288 PMC10849327 38113048

[CR12] DeFaveri J, Merilä J (2014) Local adaptation to salinity in the three-spined stickleback? J Evol Biol 27:290–302. 10.1111/jeb.12289 24330503 10.1111/jeb.12289

[CR13] Fornoni J, Valverde PL, Núñez-Farfán J (2003) Quantitative genetics of plant tolerance and resistance against natural enemies of two natural populations of *Datura stramonium*. Evol Ecol Res 5:1049–1065

[CR14] Gandon S, Michalakis Y (2002) Local adaptation, evolutionary potential and host–parasite coevolution: interactions between migration, mutation, population size and generation time. J Evol Biol 15:451–462. 10.1046/j.1420-9101.2002.00402.x

[CR15] Garrido E, Andraca-Gómez G, Fornoni J (2012) Local adaptation: simultaneously considering herbivores and their host plants. New Phytol 193:445–453. 10.1111/j.1469-8137.2011.03923.x 21988566 10.1111/j.1469-8137.2011.03923.x

[CR16] Gómez JM, Abdelaziz M, Camacho J, Muñoz-Pajares A, Perfectti F (2009) Local adaptation and maladaptation to pollinators in a generalist geographic mosaic. Ecol Lett 12:672–682. 10.1111/j.1461-0248.2009.01324.x 19453614 10.1111/j.1461-0248.2009.01324.x

[CR17] Gomulkiewicz R, Drown DM, Dybdahl MF, Godsoe W, Nuismer SL, Pepin KM, Ridenhour BJ, Smith CI, Yoder JB (2007) Dos and don’ts of testing the geographic mosaic theory of Coevolution. Heredity 98:249–258. 10.1038/sj.hdy.6800949 17344805 10.1038/sj.hdy.6800949

[CR18] Hannan Parker A, Wilkinson SW, Ton J (2022) Epigenetics: a catalyst of plant immunity against pathogens. New Phytol 233:66–83. 10.1111/nph.17699 34455592 10.1111/nph.17699

[CR19] Holeski LM, Jander G, Agrawal AA (2012) Transgenerational defense induction and epigenetic inheritance in plants. Trends Ecol Evol 27:618–626. 10.1016/j.tree.2012.07.011 22940222 10.1016/j.tree.2012.07.011

[CR20] Holsinger KE, Weir BS (2009) Genetics in geographically structured populations: defining, estimating and interpreting FST. Nat Rev Genet 10:639–650. 10.1038/nrg2611 19687804 10.1038/nrg2611 PMC4687486

[CR21] Kariñho-Betancourt E, Núñez-Farfán J (2015) Evolution of resistance and tolerance to herbivores: testing the trade-off hypothesis. PeerJ 3:e789. 10.7717/peerj.789 25780756 10.7717/peerj.789 PMC4358663

[CR22] Kawecki TJ, Ebert D (2004) Conceptual issues in local adaptation. Ecol Lett 7:1225–1241. 10.1111/j.1461-0248.2004.00684.x

[CR23] Kim J, Felton GW (2013) Priming of antiherbivore defensive responses in plants. Insect Sci 20:273–285. 10.1111/j.1744-7917.2012.01584.x 23955880 10.1111/j.1744-7917.2012.01584.x

[CR24] Lande R, Arnold SJ (1983) The measurement of selection on correlated characters. Evolution 37:1210–1226. 10.2307/2408842 28556011 10.1111/j.1558-5646.1983.tb00236.x

[CR25] Laukkanen L, Leimu R, Muola A, Lilley M, Salminen J-P, Mutikainen P (2012) Plant chemistry and local adaptation of a specialized folivore. PLoS ONE 7:e38225. 10.1371/journal.pone.0038225 22666493 10.1371/journal.pone.0038225 PMC3364215

[CR26] Leimu R, Fischer M (2008) A meta-analysis of local adaptation in plants. PLoS ONE 3:e4010. 10.1371/journal.pone.0004010 19104660 10.1371/journal.pone.0004010 PMC2602971

[CR27] Leimu R, Koricheva J (2006) A meta-analysis of genetic correlations between plant resistances to multiple enemies. Am Nat 168:E15–E37. 10.1086/505766open_in_new 16874611 10.1086/505766

[CR28] Leinonen T, Cano J, Mäkinen H, Merilä J (2006) Contrasting patterns of body shape and neutral genetic divergence in marine and lake populations of threespine sticklebacks. J Evol Biol 19:1803–1812. 10.1111/j.1420-9101.2006.01182.x 17040377 10.1111/j.1420-9101.2006.01182.x

[CR29] Leinonen T, McCairns RS, O’Hara RB, Merilä J (2013) QST-FST comparisons: evolutionary and ecological insights from genomic heterogeneity. Nat Rev Genet 14:179–190. 10.1038/nrg3395 23381120 10.1038/nrg3395

[CR30] Mauricio R, Rausher MD (1997) Experimental manipulation of putative selective agents provides evidence for the role of natural enemies in the evolution of plant defense. Evolution 51:1435–1444. 10.1111/j.1558-5646.1997.tb01467.x 28568624 10.1111/j.1558-5646.1997.tb01467.x

[CR31] Mazer S, Damuth J (2001) Nature and causes of variation. Evolutionary ecology: concepts and case studies. In: Fox CW, Roff DA and D J Fairbairn (eds), pp 3–15

[CR32] Motten AF, Antonovics J (1992) Determinants of outcrossing rate in a predominantly self-sertilizing weed, *Datura stramonium* (Solanaceae). Am J Bot 79:419–427. 10.1002/j.1537-2197.1992.tb14569.x

[CR33] Nuismer SL, Gandon S (2008) Moving beyond common-garden and transplant designs: insight into the causes of local adaptation in species interactions. Am Nat 171:658–668 18419564 10.1086/587077

[CR34] Nuñez-Farfan J, Dirzo R (1994) Evolutionary ecology of *Datura stramonium* L. in central mexico: natural selection for resistance to herbivorous insects. Evolution 48:423–436. 10.1111/j.1558-5646.1994.tb01321.x 28568306 10.1111/j.1558-5646.1994.tb01321.x

[CR35] Núñez-Farfán J, Velázquez-Márquez S, Torres-García JR, De-la-Cruz IM, Arroyo J, Valverde PL, Flores-Ortiz CM, Hernández-Portilla LB, López-Cobos DE, Matías JD (2024) A trip back home: resistance to herbivores of native and Non-Native plant populations of *Datura stramonium*. Plants 13:131. 10.3390/plants13010131 38202439 10.3390/plants13010131 PMC10780412

[CR36] Ortegón-Campos I, Parra‐Tabla V, Abdala‐Roberts L, Herrera CM (2009) Local adaptation of *Ruellia nudiflora* (Acanthaceae) to biotic counterparts: complex scenarios revealed when two herbivore guilds are considered. J Evol Biol 22:2288–2297. 10.1111/j.1420-9101.2009.01847.x 19796082 10.1111/j.1420-9101.2009.01847.x

[CR37] Parachnowitsch AL, Lajeunesse MJ (2012) Adapting with the enemy: local adaptation in plant–herbivore interactions. New Phytol 193:294–296. 10.1111/j.1469-8137.2011.04007.x 22221148 10.1111/j.1469-8137.2011.04007.x

[CR38] R Development Core Team (2011) R: a Language and environment for statistical computing. 2.15.2 Edn. R Foundation for Statistical Software. Viena, Austria

[CR39] Rice K, Jain S (1985) The ecology of natural disturbance and patch dynamics. In: Pickett S, PS W (eds) Plant population genetics and evolution in disturbed environments. Academic, New York, pp 265–286

[CR40] Shonle I, Bergelson J (2000) Evolutionary ecology of the tropane alkaloids of *Datura stramonium* L. (Solanaceae). Evolution 54:778–788. 10.1111/j.0014-3820.2000.tb00079.x 10937252 10.1111/j.0014-3820.2000.tb00079.x

[CR42] Thompson JN (2001) Coevolution. Encyclopedia of life sciences. Nature Publishing Group, London, pp 1–5

[CR41] Thompson J (2005) The geographic mosaic of Coevolution. The University of Chicago Press, Chicago

[CR43] Valverde PL, Fornoni J, Núñez-Farfán J (2001) Defensive role of leaf trichomes in resistance to herbivorous insects in *Datura stramonium*. J Evol Biol 14:424–432. 10.1046/j.1420-9101.2001.00295.x

[CR44] Vesakoski O, Jormalainen V (2013) Ignored pattern in studies of local adaptations: when the grass is greener on the allopatric site. Ideas Ecol Evol 6:32–36. 10.4033/iee.2013.6.7.n

[CR45] Villanueva-Hernández CE, Núñez-Farfán J (2025) Searching for a common host: parasitoids of *Lema daturaphila* on *Datura stramonium*. Cent Mexico PeerJ 13:e18675. 10.7717/peerj.18675 10.7717/peerj.18675 PMC11801200 39917472

[CR46] Wadgymar SM, DeMarche ML, Josephs EB, Sheth SN, Anderson JT (2022) Local adaptation: causal agents of selection and adaptive trait divergence. Annu Rev Ecol Evol Syst 53:87–111. 10.1146/annurev-ecolsys-012722-035231 37790997 10.1146/annurev-ecolsys-012722-035231 PMC10544833

[CR47] Weaver SE, Warwick SI (1984) The biology of Canadian weeds: 64. *Datura stramonium* L. Can J Plant Sci 64:979–991. 10.4141/cjps84-132

