# Supplementary material for: Local adaptation mosaic to leaf herbivores in the annual herb Datura stramonium
Source: J Plant Res. 2025 Aug 29;138(6):995–1003. doi: 10.1007/s10265-025-01664-2 (PMC12638408; doi:10.1007/s10265-025-01664-2)
Supplement: Supplementary file 1 — Supplementary Material 1 [file 10265_2025_1664_MOESM1_ESM.pdf]

Electronic supplementary materials

Title: Local adaptation mosaic to leaf herbivores in the annual herb *Datura stramonium*

Journal: Journal of Plant Research

Authors: Guillermo Castillo [1,2] , Adán Miranda-Pérez [2] , Ken Oyama [3] , Juan Núñez-Farfán [2]*

[1] Facultad de Enología y Gastronomía, Universidad Autónoma de Baja California, Carretera Transpeninsular Ensenada-Tijuana 3917, Colonia Playitas C.P. 22860 Ensenada, Baja California.

[2] Laboratorio de Genética Ecológica y Evolución, Departamento de Ecología Evolutiva, Instituto de Ecología, Universidad Nacional Autónoma de México. Circuito Exterior, Ciudad Universitaria, 14510, Distrito Federal, México.

[3] Escuela Nacional de Estudios Superiores, Universidad Nacional Autónoma de México, Campus Morelia, Michoacán, México.

Email Address: farfan@.unam.mx

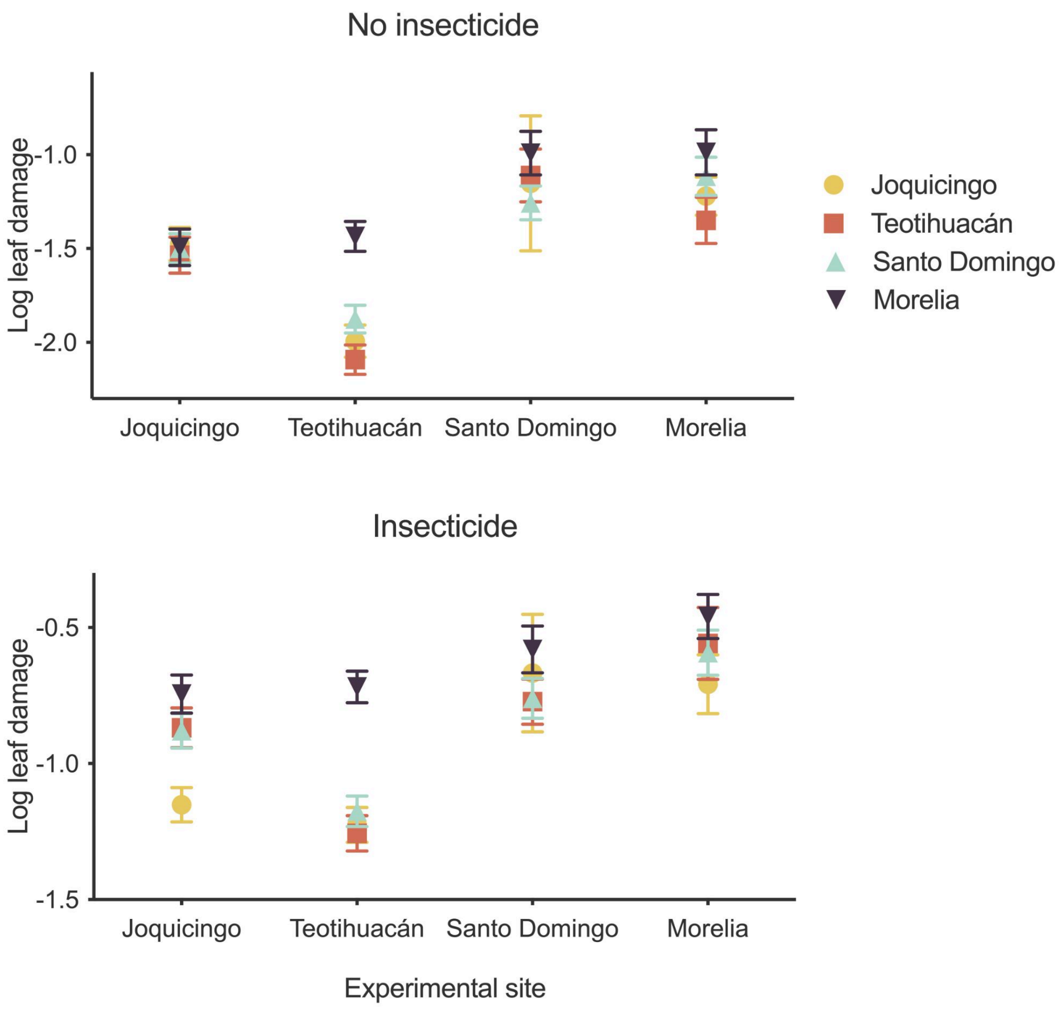

Figure S1. Mean (± SE) log-transformed leaf damage of D*atura stramonium* grown in reciprocal transplant experiments at four sites. Joquicingo (yellow circles), Teotihuacán (red squares), Santo Domingo (green triangles), and Morelia (purple inverted triangles). The top panel shows plants assigned to the "No insecticide" (Control) treatment, while the bottom panel shows plants assigned to the "Insecticide" treatment.

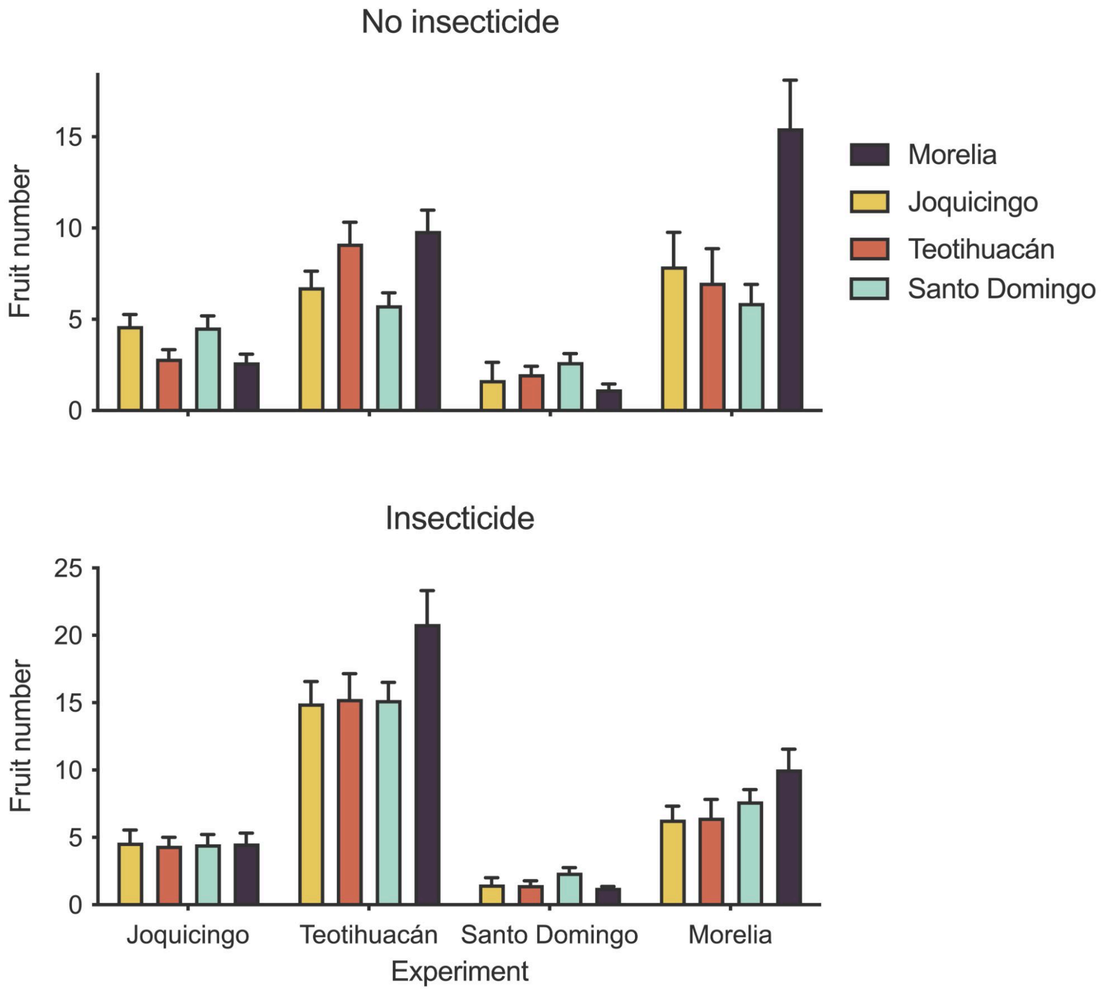

Figure S2. Mean (± SE) fruit number of *Datura stramonium* grown in reciprocal transplant experiments at four sites. Bar colors correspond to the population of origin: Morelia (purple), Joquicingo (yellow), Teotihuacán (red), and Santo Domingo (green). The top panel shows plants assigned to the “No insecticide treatment” (Control), while the bottom panel shows plants assigned to the “Insecticide” treatment.

Table S1. Geographic coordinates and habitat characteristics of populations used in the reciprocal transplant experiment.

Population	State	Vegetation type	Latitude	Longitude	Altitude (m. a. s. l.)	Mean annual Precipitation (mm^3)	Mean Temperature (°C)
Teotihuacán	México	DS	19.6871	-98.8352	2294	700	14.9
Joquicingo	México	POF	19.1153	-99.5181	2583	1000	14
Morelia	Michoacán	POF	19.6490	101.2286	1963	1000	16.4
Santo Domingo	Morelos	TDF	19.0118	-99.0627	2068	1000	16.6

DS = Desert shrub, POF = Pine-Oak forest TDF = Tropical deciduous forest.
